# Differences in prevalence of hypertension and associated risk factors in urban and rural residents of the northeastern region of the People’s Republic of China: A cross-sectional study

**DOI:** 10.1371/journal.pone.0195340

**Published:** 2018-04-05

**Authors:** Junnan Wang, Wei Sun, George A. Wells, Zhibo Li, Tianyi Li, Junduo Wu, Yangyu Zhang, Yingyu Liu, Longbo Li, Yunpeng Yu, Yihang Liu, Chao Qi, Yang Lu, Ning Liu, Youyou Yan, Lulu Liu, Gang Hui, Bin Liu

**Affiliations:** 1 Department of Cardiology, the Second Hospital of Jilin University, Changchun, Jilin, China; 2 Department of Cardiovascular Research Methods Centre, University of Ottawa Heart Institute, Ottawa, Ontario, Canada; 3 Department of Epidemiology and Biostatistics, School of Public Health, Jilin University, Changchun, Jilin, China; Nagoya University, JAPAN

## Abstract

**Background:**

Hypertension is a significant global public health problem and recognized as an important risk factor for cardiovascular diseases. This study was designed to assess the current prevalence of hypertension and to explore risk factors associated with hypertension by urban and rural status to guide the prevention and control of hypertension in Jilin province.

**Methods:**

A multi-stage stratified random cluster sampling method was used to obtain data on hypertension, which was investigated by physical examination and face-to-face questionnaire in July 2014-December 2015. Sample data were analyzed by complex weighted statistical analysis to estimate blood pressure levels and prevalence of hypertension in the province. Multivariable logistic regression analysis was used to identify factors influencing hypertension rates.

**Results:**

The prevalence of hypertension was significantly higher in rural areas than urban areas (25.93% versus 22.73%, respectively). The rates of hypertension known (46.7% versus 38.1%, respectively), control (13.7% versus 5.0%, respectively), and controlled among treated subjects (38.3% versus 17.5%, respectively) were higher in urban areas than in rural areas (all p < 0.001), while the treatment rate was not statistically significantly different between urban and rural areas (35.9% versus 28.4%, respectively). After adjusting for demographic covariates, hypertension prevalence in rural areas was still significantly greater than in urban areas (adjusted OR = 1.22; 95%CI: 1.10, 1.36; p < 0.001). Common risk factors for hypertension among urban and rural residents included older age; male; married; employed; less education; overweight/obese; greater abdominal waist circumference; family history of hypertension, stroke, or coronary heart disease; current smoker; alcohol consumption; higher visceral adiposity index; and higher body fat percentage.

**Conclusions:**

This study identified an increased risk for hypertension in rural regions of Jilin province, suggesting that rural hypertension screening and treatment guidelines should receive greater attention.

## Introduction

According to a recent report by the World Health Organization (WHO), non-communicable diseases account for 70% of all deaths worldwide, equivalent to 40 million fatalities per year [1]. Hypertension, a common risk factor for diseases such as cardiovascular, cerebrovascular, and kidney disease, has become a major risk factor for premature death and disability worldwide [1–3]. It is estimated that about 25% of adults worldwide suffer from high blood pressure, a figure that will likely increase to 29% globally by 2025 [4].

The latest *Chinese Cardiovascular Disease Report 2016* [5] indicated that the national prevalence of hypertension in individuals 18 years of age and older was 25.2%, which constitutes a dramatic increase over time compared to historical prevalence of 5.1% in 1959, 7.7% in 1980, 13.6% in 1991, and 17.6% in 2002. The *Chinese Residents Nutrition and Chronic Disease Status Report (2015)* [6] reported that the prevalence of hypertension among urban residents was 26.8% and among rural residents was 23.5%; the prevalence increased significantly with age for both urban and rural residents. The difference in prevalence of hypertension between urban and rural regions worldwide varies in both magnitude and direction [7–11]. In China, the difference in relative prevalence of hypertension in urban and rural regions has decreased, especially in the northern region [12, 13].

Jilin Province is located in the middle of the three Northeastern provinces in China. The common environmental and population traits for northern China include uniformly cold climate in winter, less active residents who prefer a high-salt diet, uneven economic development, and rapid increase in older population [14]. As a result, the burden of hypertension in Jilin Province is likely to be greater than in other provinces of China. The results of *Jilin Province residents chronic disease baseline survey (2002)*[15] showed that the overall prevalence of hypertension in adults in Jilin province aged 18 and older was 22.2%, among them the prevalence of hypertension was significantly higher in urban areas than that in rural areas (22.7% versus 21.9%, respectively). However, the results in 2012 [16] showed that the prevalence of hypertension in urban and rural areas is not significant difference (30.4% versus 30.6%, respectively). Thus, the prevalence of hypertension in Jilin Province has changed with a clear upward trend significantly in recent years, and the distribution of hypertension prevalence between urban and rural areas has changed.

An in-depth analysis of survey results from Jilin province related to risk factors for hypertension will provide an improved understanding of regional differences in hypertension prevalence, and may guide improvements in regional prevention strategies for hypertension. The aim of this study was to provide insight into the current status of hypertension in the province by urban and rural residence, and to explore risk factors associated with hypertension in relation to differences between urban and rural populations.

## Methods

### Sample

A multi-stage stratified random cluster sampling was carried out based on *The Chinese Major Cardiovascular Disease Prevalence Survey and Key Technology Research Implementation Plan* in July 2014-December 2015. This plan represents a national survey conducted by the National Cardiovascular Center and the Fuwai cardiovascular Hospital. A total of 28 provinces participated in this survey, of which Jilin Province was included as one of the pilot areas. The details of this investigation are as follows: In the first stage, eight counties (cities, districts) were randomly selected from Jilin Province using a cluster sampling method proportional to population size. In the second stage, three townships (streets) were randomly selected from each of the counties (cities, districts) sampled. In the third stage, a stratified random sample of three administrative villages (neighborhood committees, functional units) from each township (street) was selected. In the fourth stage, a simple random sampling of residents in the administrative villages (neighborhood committees, functional units) was conducted to select three villager groups (natural village) or resident groups. In the fifth stage, the cluster random sampling method was used to select the final villager group or resident group. A total of 15206 participants aged 15 years or above were participated in the study. Among them, 167 individuals failed to attend the interview, 83 individuals did not complete the questionnaire. Thus, after excluding the data for these individuals, the data for actual final sample of 14,956 participants were analyzed.

### Investigation method

A questionnaire on cardiovascular disease prevalence provided by the Chinese cardiovascular center was used. The questionnaire included family and personal health conditions, blood pressure (BP), height, weight, and education. BP was measured by standard mercury-based sphygmomanometer; measurements were repeated three times for 30s intervals and averaged for a final estimate. Investigators were trained to administer the questionnaire in a uniform and thorough fashion.

### Definition

Based on the *Chinese Hypertension Prevention Guide 2010*[17], the definition for hypertension was as follows: no current use of antihypertensive drugs and systolic BP (SBP) ≥ 140 mmHg (1 mmHg = 0.133 kPa) and/or diastolic BP (DBP) ≥ 90 mmHg, or a history of hypertension and currently using antihypertensive drugs. BP was classified as follows [17]: normal BP, SBP <120 mmHg and DBP <80 mmHg; normal high BP, SBP 120–139 mmHg and/or DBP 80–89 mmHg; grade 1 hypertension (mild), SBP 140–159 MmHg and/or DBP 90–99 mmHg; grade 2 hypertension (moderate), SBP 160–179 mmHg and/or DBP 100–109 mmHg; and grade 3 hypertension (severe), SBP ≥ 180 MmHg and/or DBP ≥110 mmHg. Known of hypertension was defined as self-report of any previous diagnosis of hypertension by a health care professional. Treatment of hypertension was defined as self-reported use of antihypertensive medications in the previous 2 weeks among those with hypertension. Control of hypertension was defined as BP < 140/90 mmHg among hypertensive participants who were under treatment.

### Quality control

All personnel involved in the investigation were trained on standards of use for mercury sphygmomanometers, weight scales, and height meters in order to minimize error between surveyors. At the time of the survey, the quality control (QC) groups that were organized by leaders of each investigation team were required to check all information after each interview. If there was missing information or logic errors, a repeated interview or examination was required on site. A second review was undertaken after the investigation day. Before data entry, the QC group conducted a third verification, and data which could not be corrected were deleted.

### Statistical analysis

SPSS 18.0 software was used for data management and statistical analysis. All estimates and analyses were weighted to represent the total population in Jilin Province aged 15 years or older. Population weights were calculated according to *The Jilin Province Census Data in 2010*[18] and the sampling age, sex, and geographic subgroups were taken into account. Continuous data were presented as mean ± standard deviation (SD) or mean with 95% confidence intervals (CI), and differences between groups were compared using analysis of variance based on complex weights. Categorical data were presented as frequency, rate, and 95% CI, and the prevalence between groups was compared using the corrected Rao-Scott chi-square test. In addition, categorical and continuous data were compared using the standardized difference, with a value <0.10 indicting no difference. Factors potentially influencing hypertension were assessed by univariate and multivariable logistic regression. The nominal significance level considered was α = 0.05.

### Ethics statement

Written informed consent was obtained from each participant before enrolment on the hypertension study. For minors, written informed consent was obtained from parents or guardians on behalf of the minors enrolled in the study. If the guardians were unable to write, then fingerprinting was used. The study was approved by the Medical Ethics Committee of the Second Hospital of Jilin University (Number 2014–006) and the Fuwai cardiovascular Hospital (Number 2012–402).

## Results

### Distribution and demographic characteristics of the study population: Urban versus rural residents

From 15,206 eligible participants, a total of 14,956 participants (6,946 males and 8,010 females; age range: 15–97 years) completed the survey. Average participant age was 41.55 ± 16.55 years, and the male to female ratio was 1.02:1. Urban residents accounted for 48.9% of participants (n = 7,307) and had an average age of 42.55 ± 16.22 years and male to female ratio of 1.02:1; the number of rural residents was 7,649, accounting for 51.1% of the participants, with an average age of 40.96 ± 16.71 years and male to female ratio of 1.03:1. The demographic characteristics of the residents and distribution of physical indicators in urban and rural areas are shown in Table 1.

**Table 1:** Distribution and demographic characteristics of the study population.

Characteristic	Overall N (%) / Mean ± SD	Urban N (%) / Mean ± SD	Rural N (%) / Mean ± SD	*P*	Standardized difference
Age	41.55 ± 16.55	42.55 ± 16.22	40.96 ± 16.71	<0.001	0.097
Age group				<0.001	
15~44	7545 (50.4)	4009 (54.9)	3536 (46.2)		0.175
45~64	4447 (29.7)	1917 (26.2)	2530 (33.1)		0.152
65+	2964 (19.8)	1381 (18.9)	1583 (20.7)		0.045
Gender				0.68	
M	6946 (50.63)	3366 (50.39)	3580 (50.78)		0.008
F	8010 (49.37)	3941 (49.61)	4069 (49.22)		0.008
Ethnicity					
Han	14174 (94.43)	7003 (96.82)	7171 (93)	<0.001	0.174
Others	782 (5.57)	304 (3.18)	478 (7)		0.174
Education				<0.001	
None	883 (3.65)	258 (2.15)	625 (4.55)		0.134
Primary level	8809 (63.34)	3584 (54.39)	5225 (68.7)		0.297
Secondary level	3121 (20.49)	1952 (23.97)	1169 (18.4)		0.137
Tertiary level	2143 (12.52)	1513 (19.49)	630 (8.35)		0.326
BMI				0.68	
Underweight	702 (4.58)	354 (4.55)	348 (4.6)		0.002
Normal	8851 (59.4)	4225 (59.1)	4626 (59.59)		0.010
Overweight	4473 (29.78)	2251 (30.35)	2222 (29.44)		0.020
Obese	930 (6.24)	477 (6.01)	453 (6.38)		0.015
Smoking				<0.001	
Never	11778 (75.85)	6024 (78.25)	5754 (74.42)		0.090
Former	252 (1.41)	108 (1.3)	144 (1.48)		0.015
Current	2926 (22.73)	1175 (20.46)	1751 (24.1)		0.088
Alcohol	2299 (17.62)	1056 (17.98)	1243 (17.41)	0.46	0.009
BMI	24.01 ± 0.04	24.04 ± 0.05	24.00 ± 0.05	0.55	0.011
Basal metabolism	1397.47 ± 2.42	1400.2 ± 3.34	1395.84 ± 3.3	0.35	0.018
Body Fat	26.20 ± 0.08	26.61 ± 0.12	25.95 ± 0.11	<0.001	0.077
VAI	8.54 ± 0.05	8.37 ± 0.06	8.64 ± 0.07	0.003	0.056
SBP	128.91 ± 0.16	126.18 ± 0.24	130.55 ± 0.22	<0.001	0.247
DBP	76.81 ± 0.10	75.96 ± 0.13	77.32 ± 0.14	<0.001	0.133

BMI: body mass index, M: male, F: female, VAI: Visceral Adiposity Index, SBP: Systolic blood pressure, DBP: Diastolic blood pressure.

### Prevalence of hypertension in urban and rural residents

The prevalence of hypertension in the adult population aged 15 and older in 2015 was 24.73%. The prevalence among adult urban residents was significantly lower than among adult rural residents (22.73% vs. 25.93%; p < 0.001). The prevalence of hypertension in rural residents aged 15 to 64 was higher than for urban residents (p < 0.001), and the prevalence of hypertension in rural males was higher than in urban males (p < 0.001). The prevalence of hypertension in the Han, overweight, never smokers, current smokers, alcohol consumers, and non-drinking populations of rural residents was higher than for the same subgroups in urban residents; all differences were statistically significant (p < 0.05) (Table 2).

**Table 2:** Prevalence of hypertension in urban and rural residents.

Characteristic	Overall	Urban	Rural	*P*
	% (95% CI)	% (95% CI)	% (95% CI)	
All	24.73(23.95, 25.54)	22.73 (21.60, 23.90)	25.93 (24.88, 27.01)	<0.001
Age group				
15~44	10.53 (9.66, 11.39)	8.08 (7.04, 9.13)	11.92 (10.70, 13.12)	<0.001
45~64	39.68 (38.10, 41.26)	35.23 (32.72, 37.74)	42.47 (40.45, 44.50)	<0.001
65~	62.44 (60.44, 64.45)	63.08 (60.05, 66.11)	61.99 (59.33, 64.66)	0.60
Gender				
Male	25.60 (24.42, 26.81)	22.66 (21.02, 24.40)	27.34 (25.76, 28.99)	<0.001
Female	23.84 (22.82, 24.90)	22.80 (21.28, 24.39)	24.47 (23.12, 25.89)	0.117
Ethnicity				
Han	24.77 (23.96,25.59)	22.88 (21.72, 24.07)	25.95 (24.86, 27.07)	<0.001
Non Han	24.13 (20.87,27.72)	18.35 (13.54, 24.39)	25.70 (21.81, 30.01)	0.043
Education				
None	49.84 (45.85, 53.83)	54.48 (46.16, 62.56)	48.52 (43.99, 53.08)	0.217
Primary level	30.23 (29.15, 31.32)	30.25 (28.53, 32.03)	30.22 (28.87, 31.60)	0.973
Secondary level	12.22 (10.93, 13.63)	13.62 (11.80, 15.67)	11.12 (9.39, 13.12)	0.069
Tertiary level	10.09 (8.60, 11.81)	9.44 (7.74, 11.47)	11.00 (8.49, 14.16)	0.354
BMI				
Underweight	8.00 (6.20, 10.27)	6.74 (4.29, 10.44)	8.75 (6.40, 11.84)	0.343
Normal	18.05 (17.17, 18.97)	16.36 (15.09, 17.71)	19.06 (17.89, 20.29)	0.003
Overweight	35.74 (34.11, 37.39)	33.27 (30.95, 35.69)	37.26 (35.07, 39.50)	0.017
Obese	48.09 (44.27, 51.94)	44.28 (38.80, 49.90)	50.25 (45.13, 55.36)	0.123
Smoking				
Never	21.95 (21.11, 22.82)	20.65 (19.46, 21.90)	22.77 (21.63, 23.96)	0.014
Former	53.10 (45.66, 60.41)	53.42 (41.29, 65.16)	52.93 (43.58, 62.09)	0.950
Current	32.24 (30.35, 34.19)	28.73 (25.84, 31.81)	34.02 (31.60, 36.53)	0.008
Alcohol				
No	22.62 (21.80, 23.45)	21.42 (20.23, 22.65)	23.33 (22.24, 24.45)	0.023
Yes	34.63 (32.41, 36.92)	28.73 (25.62, 32.06)	38.28 (35.29, 41.37)	<0.001

### Known, treatment, and control of hypertension in urban and rural residents

The rates of hypertension known, treatment, control, and controlled among treated subjects were 42.3%, 31.7%, 8.8% and 27.9%, respectively. The rates of hypertension known (46.7% versus 38.1%, respectively), control (13.7% versus 5.0%, respectively), and controlled among treated subjects (38.3% versus 17.5%, respectively) were higher in urban areas than in rural areas (all p < 0.001), while the treatment rate was not statistically significant in urban versus rural areas (35.9% versus 28.4%, respectively) (Table 3).

**Table 3:** The known, treatment and control of hypertension in urban and rural residents.

Hypertensive cases	Total(n = 4332)	urban(n = 1913)	rural(n = 2419)	*P*-value
known	1833(42.3)	911(46.7)	922(38.1)	<0.001
Treatment	1373(31.7)	687(35.9)	686(28.4)	0.65
Control	383(8.8)	263(13.7)	120(5.0)	<0.001
Controlled among treated subjects	383/1373(27.9)	263/687(38.3)	120/686(17.5)	<0.001

### Unadjusted and adjusted relationship of hypertension in urban and rural residents

**Fig 1:**
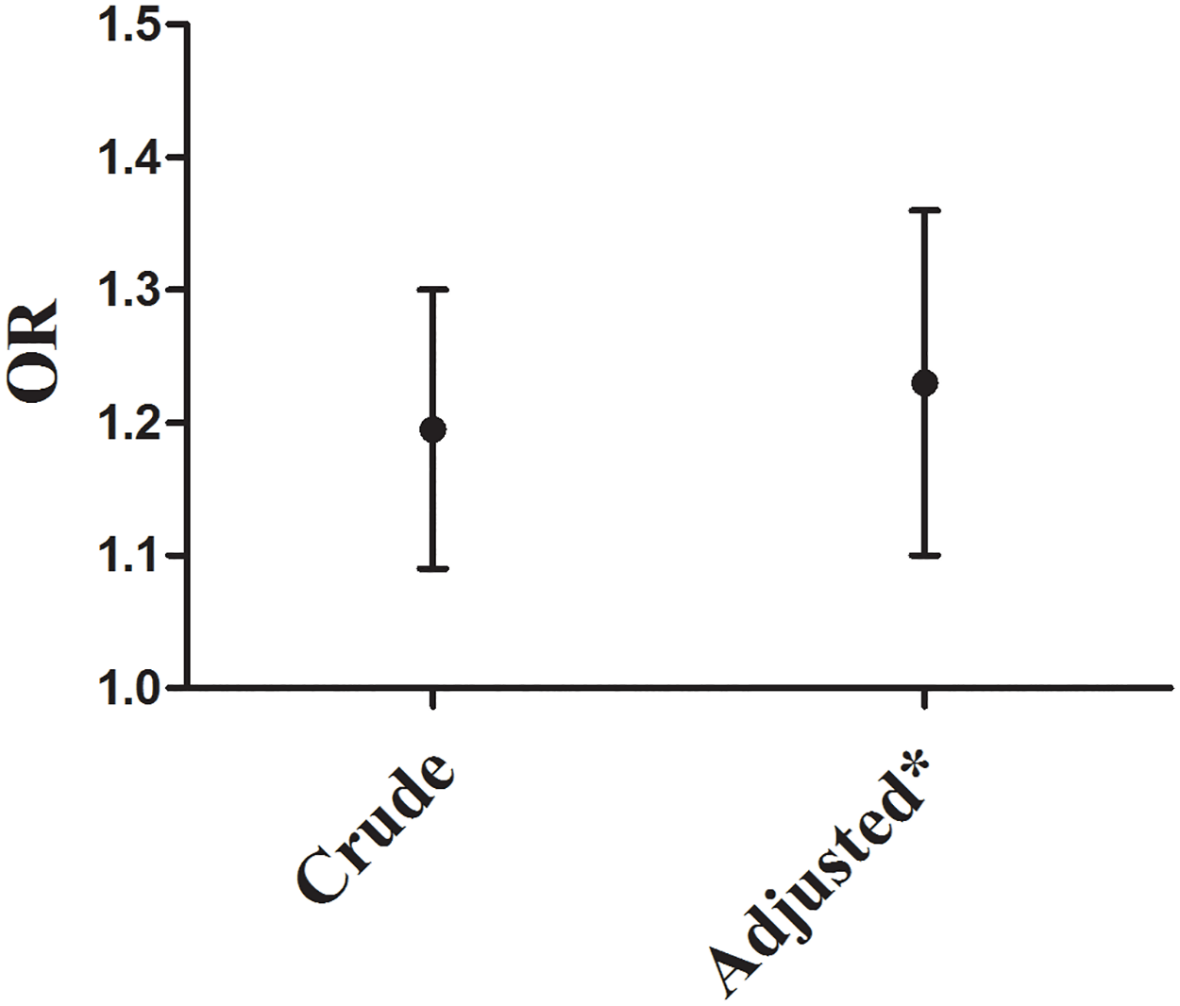
Unadjusted and adjusted relationship of hypertension to urban/rural. * Adjusted for gender, region, age, education level, retired status, marital status, BMI, Family of hypertension.

An unadjusted logistic regression model indicated that the risk of hypertension for rural residents was greater than that for urban residents (OR = 1.19, 95%CI: 1.09, 1.30; p < 0.001). A multivariable logistic regression model was used to adjust for potential confounders. After adjusting for sex, age, education level, retired status, marital status, body mass index (BMI), and family history of hypertension, the risk of hypertension in rural areas was significantly greater than that in urban areas (adjusted OR = 1.22, 95%CI: 1.10, 1.36; p < 0.001). Results are shown in Fig 1.

### Factors associated with the prevalence of hypertension in urban and rural residents

Multivariable logistic regression analysis results showed that common risk factors of hypertension for urban and rural residents included older age; male; married; less education; overweight/obese; greater abdominal waist circumference (AWC); family history of hypertension, stroke, or coronary heart disease (CHD); current smoker; alcohol consumer; higher visceral adiposity index (VAI); and higher body fat percentage (BFP). A protective factor for urban and rural residents was being unemployed (versus employed); retired and student status (versus employed) were protective factors only in urban areas. Detailed results are provided in Table 4.

**Table 4:** Factors associated with the prevalence of hypertension in urban and rural residents by multivariate logistic regression models.

	Urban		*P*-value	Rural		*P*-value
	Crude OR (95%CI)	Adjusted* OR (95%CI)		Crude OR (95%CI)	Adjusted* OR (95%CI)	
Age (ref: 15~44)						
45~64	5.59 (4.99, 6.26)	4.30(3.78,4.88)	<0.001	5.46(4.74,6.29)	4.32(3.68,5.08)	<0.001
65~	14.13 (12.47, 16.02)	12.45(10.6,14.49)	<0.001	12.06(10.26,14.18)	11.34(9.29,13.84)	<0.001
Gender (ref: Female)	1.10 (1.01, 1.2)	1.26(1.13,1.39)	0.03	1.16(1.04,1.3)	1.3(1.14,1.48)	0.008
Race (ref: Han)	0.97 (0.8, 1.17)	0.98(0.77,1.23)	0.72	0.99(0.79,1.23)	0.98(0.75,1.28)	0.908
Employment (ref: Employed)						
Retired	2.67 (2.23, 3.21)	0.77(0.61,0.98)	<0.001	2.57(1.59,4.16)	1.06(0.59,1.89)	0.712
Student	0.11 (0.08,0.16)	0.60(0.40,0.92)	<0.001	0.12(0.08,0.17)	0.69(0.4,1.21)	0.508
Unemployed	1.18 (1.07,1.31)	0.86(0.76,0.96)	<0.001	1.01(0.89,1.15)	0.75(0.64,0.87)	<0.001
Marital (ref: Married)	0.15 (0.12,0.18)	0.57(0.45,0.71)	<0.001	0.14(0.11,0.18)	0.56(0.41,0.76)	<0.001
Education level (ref: College or higher)						
Illiterate	8.85 (6.98,11.22)	2.92(2.18,3.90)	<0.001	7.62(5.42,10.71)	2.91(1.95,4.33)	<0.001
Primary	3.86 (3.21,4.64)	2.25(1.83,2.77)	<0.001	3.5(2.61,4.7)	2.38(1.72,3.31)	<0.001
Middle	1.24(1,1.54)	1.24(0.98,1.58)	0.694	1.01(0.72,1.43)	1.4(0.96,2.04)	0.782
BMI (ref: Normal)						
Overweight	2.52(2.3,2.77)	2.30(2.06,2.58)	<0.001	2.52(2.23,2.85)	2.42(2.09,2.79)	<0.001
Obese	4.21(3.56,4.96)	5.11(4.16,6.27)	<0.001	4.29(3.44,5.34)	5.37(4.11,7.01)	<0.001
AWC (ref:<90M, <85F)						
≥90M, ≥85F	2.33(2.06,2.62)	1.38(1.19,1.60)	<0.001	2.14(1.83,2.51)	1.21(0.99,1.47)	0.096
≥95M, ≥90F	3.47(3.12,3.87)	1.71(1.46,2.00)	<0.001	3(2.61,3.46)	1.34(1.1,1.65)	<0.001
Family history of hypertension	2.27(2.04,2.53)	2.40(2.10,2.73)	<0.001	2.16(1.87,2.48)	2.16(1.83,2.56)	<0.001
Family history of stroke	4.79(3.2,7.16)	1.98(1.21,3.22)	<0.001	5.08(3.07,8.42)	2.27(1.22,4.22)	<0.001
Family history of CHD	4.54(3.87,5.33)	1.73(1.40,2.14)	<0.001	4.12(3.34,5.09)	1.72(1.31,2.27)	<0.001
Smoker (ref: No)						
Former	4.41(2.70,7.20)	1.74(0.95,3.20)	0.468	3.84(2.61,5.57)	1.55(0.97,2.48)	0.392
Current	1.55(1.32,1.82)	1.52(1.22,1.88)	<0.001	1.75(1.54,1.99)	1.27(1.08,1.50)	<0.001
Alcohol (ref: No)	1.81(1.62,2.02)	1.49(1.28,1.73)	<0.001	2.04(1.77,2.35)	1.48(1.22,1.8)	<0.001
VAI (ref: <10)						
10~14	2.78(2.51,3.07)	1.47(1.29,1.68)	<0.001	2.69(2.36,3.07)	1.32(1.11,1.57)	<0.001
15~30	5.39(4.72,6.17)	2.25(1.86,2.73)	<0.001	4.7(3.97,5.58)	1.82(1.44,2.31)	<0.001
BFP (ref: <10M, <20F)						
10~19M, 20~29F	1.24(0.96,1.61)	0.98(0.73,1.30)	0.159	1.2(0.89,1.64)	0.9(0.64,1.25)	0.273
20~24M, 30~34F	2.91(2.25,3.76)	1.33(0.99,1.77)	0.082	3.07(2.27,4.16)	1.28(0.91,1.8)	0.094
≥25M, ≥35F	5.93(4.61,7.62)	1.64(1.22,2.20)	<0.001	5.9(4.38,7.95)	1.46(1.03,2.08)	<0.001

BMI: body mass index. AWC: Abdominal waist circumference. CAD: coronary heart disease. M: male. F: female. VAI: Visceral Fat. BFP: body fat percentage.

* Adjusted for gender, region, age, education level, retired status, marital status, BMI, Family of hypertension.

## Discussion

This study found that the overall prevalence of hypertension in adults in Jilin province aged 15 and older was 24.73%, comparable to the national prevalence of 25.5%[6]. The prevalence of hypertension was significantly higher in rural areas compared with urban areas (25.93% versus 22.73%, respectively); which was contrary to national prevalence estimates in China based on a 2015 national survey showing a lower prevalence in rural regions than urban regions (23.5% versus 26.8%, respectively)[6]. However, national data show that the prevalence gap between urban and rural areas has gradually narrowed. In 1959, the national prevalence of hypertension in urban areas was 1.5 times the prevalence in rural areas [19]. By 2002, the urban-rural ratio was much diminished (urban-to-rural: 1.2)[20] and by 2015 it was as low as 1.14 [6]. In some regions of China, like Jiangxi province [21], results showed the prevalence of hypertension in rural areas is lower than in urban areas (24.0% versus 33.7%, respectively), while other regions, like Zhejiang (25.2% versus 24.1%, respectively)[22], Shanxi province (22.2% versus 20.7%, respectively)[23], and Shandong province (24.6% versus 20.8%, respectively)[24] demonstrated contrary results. In recent studies from Turkey [11] and Malaysia [9], the prevalence of hypertension in rural areas was higher than in urban areas, demonstrating a similar trend to Jilin province. Furthermore, the prevalence of hypertension in rural areas in the Jilin province is higher than that in rural regions at the national level based on a meta-analysis (25.93% versus 22.8%, respectively)[25]. Our epidemiological data suggest that the gap in the hypertension rate between rural and urban areas increased, which is contrary to results from a 2012 survey [13] in the Jilin Province.

Common risk factors for hypertension include age, gender, smoking, drinking, physical exercise, sleep time and quality, diet, overweight and obesity, among others [26–28]. Due to the significant differences in cultural practices, living habits, living environments, and sources of information between urban and rural areas, the prevalence of common risk factors of hypertension in urban and rural areas is also different. The 2009 national survey [29] showed that the smoking rate of urban residents in China is 30.09%, which is lower than 31.55% in rural areas. Lv et al.[30] found no significant differences in alcohol consumption between urban and rural areas in Jiangsu Province, but the heavy drinking rate among rural residents was significantly higher than that of urban areas (17.75% vs 15.69%). Zhang et al.[31] found that the duration and frequency of physical exercise for rural residents were significantly lower than those for urban residents. However, most of the current studies did not consider the time and intensity of rural residents’ participation in agricultural work, Therefore, we cannot accurately judge the differences in physical activity level between urban and rural residents. The study also found that rural residents had significantly better sleep quality and sleep quality than urban residents. Epidemiological surveys carried out by Henan Province in 2012 [32] showed that the obesity rate of urban residents was significantly higher (15.54%) than that of rural residents (12.95%), that of urban males was higher than that of females, and that of rural females was higher than that of males. Studies in Guangxi Province [33] and Tianjin [34] all support that urban obesity rates are higher than those in rural areas. Worldwide, the differences between urban and rural areas of risk factors in different countries are also different. In Poland, the prevalence of obesity in rural hypertension patients is significantly higher than that in urban areas [35]; the prevalence of obesity in rural areas in the Mediterranean is also higher than that in urban areas [36]. Rural residents also have higher alcohol consumption rates than urban residents, but smoking rates are now higher in urban areas than in rural areas.

Our study showed that in urban areas, compared with being employed, retired individuals and students had much lower prevalence of hypertension. Retired individuals and students might have better knowledge of hypertension prevention. However, a study in 13 European countries [37] found no association between not working and hypertension. The investigators of the Malaysian study [9] indicated that higher prevalence of hypertension in the rural population could be explained by lifestyle factors such as lack of physical activity, excess dietary intake of sodium and fat, as well as higher rates of obesity, which have spread from urban to rural areas at an alarming rate. The investigators of the Turkish [11] study found urbanization to be a contributing factor to hypertension rates in a multivariate regression analysis. Urbanization influences lifestyle patterns, leading to a decrease in physical activity, changes in food consumption, and increased stress. Finally, the rural population in the Jilin Province prefer a high-salt diet [14]. A study from the Shandong province [24] suggested that dietary salt intake is high, especially in rural areas, and has not changed much in the province over the past 10 years. Other risk factors for hypertension included age, overweight, obesity [38–40] and modifiable lifestyle factors, such as alcohol consumption [41, 42] and smoking [43, 44], which are consistent with previous studies.

In our study, the level of education is a protective factor of hypertension. Rural residents may lack knowledge about maintaining good health. Lower levels of education for rural adults may lead to unhealthy lifestyles and lack of knowledge regarding the prevention of hypertension compared to urban adults. However, Lei et al. [45] found no significant education gradients in the actual prevalence of hypertension. A significant education gradient exists in the awareness, treatment and control of hypertension although no gradient is found in actual prevalence in urban, but not in rural areas. Tian et al.[46] and Wu et al.[47] reported that increased awareness and treatment were found among those with higher education levels. However, Wang et al.[48] and a multi-ethnic Asian population study [49] reported that high education levels were associated with poor hypertension awareness and treatment.

Our study also found that marriage is a common risk factor for hypertension in urban and rural areas. In this regard, we suspect that married people may face much more pressures such as childbirth. Some studies indicate that marital transition, which involves lifestyle changes, may negatively affect physical health and increase the risks for certain diseases [11, 50]. However, others show that married subjects were more protected against high BP compared to others marital status, possibly be because of the support from the partner [10, 51]. However, further statistical analyses about the impact of marriage on hypertension are required.

Furthermore, lower levels of known, treatment, and control rate of hypertension have become another challenge in Chinese rural areas, especially in Jilin province. The national hypertension known rates in rural and urban areas in 1999, 2002, and 2014 were 13.5% vs. 35.6%, 22.5% vs. 41.1%,[25] 39.9% vs.50.9%,[52] respectively. Treatment rates of hypertension in rural and urban areas in 1999, 2002, and 2014 were 13.9% vs. 35.6%, 17.4% vs. 35.1%,[25] and 30.1% vs. 46.7%,[52] respectively. Rates of BP control in urban and rural areas in 1998, 2002, and 2014 were 4.4% vs. 2.6%, 9.7% vs. 3.5%,[25] and 30.1% vs. 46.7%,[52] respectively. Earlier research in Dehui City (Jilin province BP control pilot sites) showed that the awareness (22.34% versus 20.96%, respectively), treatment (16.55% versus 13.75%, respectively) and control (0.83% versus 1.55%, respectively) rate was not statistically significant in urban versus rural areas [14]. Our results were significantly lower than the national average. Interestingly, the difference in treatment rates between urban and rural areas was not statistically significant. We presume that the expanded coverage of Chinese health insurance may help improve the treatment rate among rural residents [53]. Despite great improvements, rates of hypertension known and control in rural areas is still significantly lower than in urban areas. In addition, there is still an obvious gap in rates of known, treatment, and control of hypertension between China and the developed countries [54, 55].

There are some limitations to this research. First, we measured blood pressure only during a single visit, which may not yield a fully accurate estimation of the true prevalence of hypertension. Second, due to economic and human resource constraints, some data, including physical activity, salt intake, levels of homocysteine, blood lipid levels, and blood glucose levels, were lacking. Third, although we used a unified standard of hypertension diagnosis, the time of blood pressure measurement and white coat syndrome may have impacted the identification of hypertension. Fourth, this study only conducted an investigation in Jilin province, and while the results can be generalized to the northeast region, they cannot be generalized to the entire country.

The rapid and disproportional economic growth in the northeastern region of China has brought unexpected changes from a public health perspective, with the greatest impacts on health in rural areas. This study provides data on current hypertension rates and factors affecting these rates in the Jilin province, which may guide the development of practical and effective strategies for managing and preventing hypertension, especially in rural areas.
